# Complete mitochondrial genome of the *Pasiphila chloerata* (Lepidoptera: Geometridae) and its phylogenetic implications

**DOI:** 10.1080/23802359.2019.1692725

**Published:** 2019-11-20

**Authors:** Lu Song, Yu-Xia Shi, Jun-Hao Li, Hong-Fei Zhang, Wei-Li Ding, Jin Li, Ming-Sheng Yang

**Affiliations:** College of Life Science and Agronomy, Zhoukou Normal University, Zhoukou, PR China

**Keywords:** Geometroidea, mitochondrial genome, moths, phylogeny

## Abstract

In this study, the complete mitochondrial genome of *Pasiphila chloerata* (Mabille) was sequenced and its phylogenetic implications were investigated. The *P. chloerata* mitogenome is a circular, double-stranded molecule, with 15,602 bp in length. The typical 37 mitochondrial genes (13 protein-coding genes (PCGs), 22 tRNAs, and 2 rRNAs) and an A + T-rich region are included. Gene content and arrangement are highly conserved and typical of Lepidoptera. Phylogenetic analyses based on the combined 37 mitochondrial genes consistently recovered the Larentiinae and Ennominae involved are reciprocally monophyletic with the highest supports. The *P. chloerata* was clustered with other two members of the Larentiinae, reinforcing that of previous morphological studies.

The Geometridae, with more than 26,000 described species, is one of the most speciose groups in Lepidoptera (van Nieukerken et al. 2011; Liu et al. 2014). However, mitochondrial genomes (mitogenomes) of only 12 geometrid species have been reported and 10 of them were from the Ennominae. Mitogenome sequences contain high genetic information (Timmermans et al. 2014) and more taxa with mitogenomes sequenced would effectively facilitate evolutionary studies on this group. In this study, the complete mitogenome of an additional geometrid species were sequenced using next-generation sequencing. The *Pasiphila chloerata* (Mabille, 1870), belonging to the Larentiinae, is widely distributed in the Palearctic region.

Adult specimens were collected from Mountain Jigongshan (114°06′ 56′′E, 31°49′25′′N) of Henan Province, China. After species identification and the extraction of genomic DNA, one library was constructed, and an Illumina Miseq platform (Illumina, San Diego, CA) was used for sequencing with the strategy of 250 paired-ends. Voucher specimens are deposited in the Biology Laboratory of Zhoukou Normal University (accession number: 2018JGSA6 and 2018JGSA14), China.

The *P. chloerata* mitogenome (GenBank accession number: MN598218) is a circular, double-stranded molecule, with 15,602 bp in length, and includes typical 37 mitochondrial genes (13 PCGs, 22 tRNAs, and 2 rRNAs) plus an A + T-rich region. Gene content and arrangement are highly conserved and typical of Lepidoptera. The nucleotide composition is A 40.11%, G 7.79%, C 11.57, and T 40.53, showing a highly A/T bias as commonly present in insects (Boore 1999).

The total length of 13 protein-coding gene (PCGs) of *P. chloerata* is 11,197 bp, encoding 3731 amino acids. Most PCGs use the conventional ATN as start codon, with an exception being CGA for the *cox1*. TAA is routinely used as stop codon, whereas the incomplete termination codon T is recognized in *cox1*, *cox2*, *nad5*, and *nad4*. Typically, 22 tRNAs are recognized. All tRNAs exhibit typical clover-leaf secondary structure, but *trnS1* (AGN) lacks the DHU arm, which is common in Lepidoptera insects (Garey and Wolstenholme 1989). Two rRNA genes, *rrnS* and *rrnL* were recognized. The lengths are 781 bp and 1381 bp, respectively. There are ten overlapping regions ranging from 1 to 8 bp. A large intergenic region (50 bp) between *trnQ* and *nad2* was recognized and this sequence even is regarded as an autapomorphy of Lepidoptera (Cao et al. 2014). Besides, the intergenic region (18 bp) between the *trnS2* and *nad1* was also recognized as characterized by the presence of the ‘ATACTAA.’ As the largest non-coding region, the A + T-rich region contains typical conserved sequence elements such as the motif ‘ATAGA’ and subsequent poly-T structure.

Phylogenetic trees were constructed based on a dataset including all 37 mitochondrial genes of the *P. chloerata* sequenced herein together with all other 12 geometrid species and one epicopeiid species in Geometroidea. Both maximum likelihood and Bayesian inference analyses (Figure 1) consistently recovered the Larentiinae and Ennominae involved were reciprocally monophyletic with the highest supports. The *P. chloerata* was clustered with other two members of the Larentiinae, reinforcing that of previous morphological studies.

**Figure 1.**
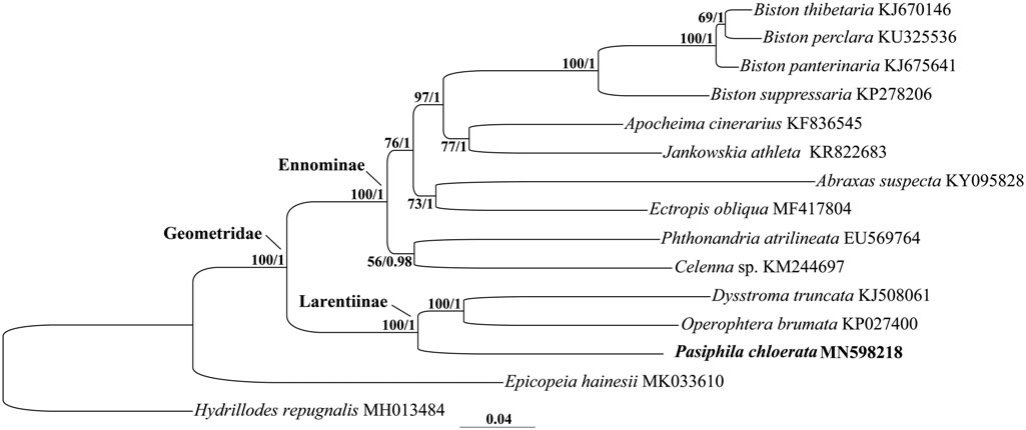
Phylogenetic tree obtained from Bayesian analysis based on the dataset consisting of all 37 mitochondrial genes. The species with newly sequenced mitogenome was emphasized in bold. Numbers separated by a slash on node are bootstrap value for maximum-likelihood analysis and posterior probability for Bayesian analysis.
